# Lentiviral Vector-Mediated Gradients of GDNF in the Injured Peripheral Nerve: Effects on Nerve Coil Formation, Schwann Cell Maturation and Myelination

**DOI:** 10.1371/journal.pone.0071076

**Published:** 2013-08-12

**Authors:** Ruben Eggers, Fred de Winter, Stefan A. Hoyng, Kasper C. D. Roet, Erich M. Ehlert, Martijn J. A. Malessy, Joost Verhaagen, Martijn R. Tannemaat

**Affiliations:** 1 Laboratory for Neuroregeneration, Netherlands Institute for Neuroscience, an institute of the Royal Academy of Arts and Sciences, Amsterdam, The Netherlands; 2 Department of Neurosurgery, Leiden University Medical Center, Leiden, The Netherlands; 3 Department of Molecular and Cellular Neurobiology, Center for Neurogenomics and Cognition Research, Vrije Universiteit Amsterdam, Amsterdam, The Netherlands; 4 Department of Neurology, Leiden University Medical Center, Leiden, The Netherlands; Biological Research Centre of the Hungarian Academy of Sciences, Hungary

## Abstract

Although the peripheral nerve is capable of regeneration, only a small minority of patients regain normal function after surgical reconstruction of a major peripheral nerve lesion, resulting in a severe and lasting negative impact on the quality of life. Glial cell-line derived neurotrophic factor (GDNF) has potent survival- and outgrowth-promoting effects on motoneurons, but locally elevated levels of GDNF cause trapping of regenerating axons and the formation of nerve coils. This phenomenon has been called the “candy store” effect. In this study we created gradients of GDNF in the sciatic nerve after a ventral root avulsion. This approach also allowed us to study the effect of increasing concentrations of GDNF on Schwann cell proliferation and morphology in the injured peripheral nerve. We demonstrate that lentiviral vectors can be used to create a 4 cm long GDNF gradient in the intact and lesioned rat sciatic nerve. Nerve coils were formed throughout the gradient and the number and size of the nerve coils increased with increasing GDNF levels in the nerve. In the nerve coils, Schwann cell density is increased, their morphology is disrupted and myelination of axons is severely impaired. The total number of regenerated and surviving motoneurons is not enhanced after the distal application of a GDNF gradient, but increased sprouting does result in higher number of motor axon in the distal segment of the sciatic nerve. These results show that lentiviral vector mediated overexpression of GDNF exerts multiple effects on both Schwann cells and axons and that nerve coil formation already occurs at relatively low concentrations of exogenous GDNF. Controlled expression of GDNF, by using a viral vector with regulatable GDNF expression, may be required to avoid motor axon trapping and to prevent the effects on Schwann cell proliferation and myelination.

## Introduction

Peripheral nerve injury has a severe and lasting negative impact on the quality of life. Although the peripheral nerve is capable of regeneration, only a small minority of patients regain normal function after surgical reconstruction of a major peripheral nerve lesion. The distance between the site of nerve repair and the distal end organs (i.e., the distance regenerating axons must bridge in order to reinnervate their target cells) is a major determinant of functional outcome [1]. This commonly held view is supported by clinical observations: in injuries of the proximal upper limb or the brachial plexus, the functional outcome of surgical intervention is particularly unsatisfactory [2]. Furthermore, after surgical treatment of brachial plexus injuries, innervation of proximal targets (e.g., the biceps muscle) is common, but recovery of hand function almost never occurs [2].

The lumbar ventral root avulsion model in the rat is unique because it mimics the essential characteristics of proximal nerve lesions in humans: poor reinnervation of distal targets and only minimal recovery of function [3]. Following reimplantation of the avulsed roots in the spinal cord, motor axons traverse the inhibitory environment of the CNS and enter the nerve roots [3]–[5]. However, over the course of several months, neurotrophic factor production in the distal nerve segments decreases [6]. The number of axons reaching the distal part of the nerve is very low, target muscles lose up to 90% of their original weight and functional recovery is virtually non-existent [3]. Lumbar ventral root avulsion followed by reimplantation of the severed roots is therefore an excellent model to test the efficacy of regenerative treatments for the injured peripheral nerve, including gene and cell-based treatment strategies.

Several neurotrophic factors have been shown to stimulate neurite outgrowth after viral vector-mediated overexpression in the injured peripheral nerve [7]. In particular glial cell-line derived neurotrophic factor (GDNF) has potent survival- and outgrowth-promoting effects on motoneurons [8], [9]. Lentiviral vector-mediated overexpression of GDNF prevents motoneuron atrophy and enhances axonal outgrowth into reimplanted nerve roots in a ventral root avulsion model [10]. Unfortunately, locally elevated levels of GDNF cause trapping of regenerating axons and actually reduce the number of axons extending more distally [11], [12]. This phenomenon has been called the “candy store” effect [13].

It is not known how GDNF causes trapping of axons in the peripheral nerve. It has been hypothesized that GDNF, as a target-derived neurotrophic factor, has a neurotropic effect, acting as a directional cue on regenerating axons [11], [14]. Several observations of the actions of GDNF and other neurotrophic factors in vitro and in vivo support this hypothesis. (i) Gradients of brain-derived neurotrophic factor (BDNF) can affect the outgrowth and turning behaviour of hippocampal neurons *in vitro* [15]. (ii) BDNF and GDNF gradients appear to direct the outgrowth of axons in the injured striatum, since axons continue to grow until they reach the point of maximal neurotrophic factor expression [16]. (iii) In a rat sciatic nerve injury model, the number of regenerated axons in artificial conduits with GDNF-loaded microspheres releasing the highest GDNF concentration at the distal end was higher than that in nerve guides with uniformly distributed GDNF release, although this may have been caused by increased axonal sprouting [17]. (iv) In a rodent spinal cord injury model, the injection of a Lentiviral vector expressing BDNF rostral to the injury generated a BDNF-gradient and promoted directional growth of axons [18]. In a similar model, gradients of Neurotrophin 3 (NT-3) also appeared to attract regenerating axons, and this approach stimulated outgrowth over a relatively short distance in the spinal cord [19]. Finally, (v) in transgenic mice, regeneration after nerve crush injury is enhanced when GDNF is overexpressed in target muscles, whereas overexpression of GDNF in the nerve and the spinal cord delays regeneration [20]. These studies suggest that if regrowing axons in the peripheral nerve are trapped by a neurotropic effect exerted by high local levels of GDNF, it might be overcome by applying a gradient that stimulates axons to continue to extend towards increasing GDNF levels.

However, it is also possible that axon trapping is caused by a different mechanism that is not related to the neurotropic effect of GDNF. GDNF could have direct effects on Schwann cells [21], on the interactions of Schwann cells with axons or on myelination, a process known to be influenced by GDNF [22].

In this study we created gradients of GDNF in the sciatic nerve after a ventral root avulsion to test the hypothesis that this will prevent trapping of regenerating axons. This approach also allowed us to study the effect of increasing concentrations of GDNF on Schwann cells in the injured peripheral nerve.

## Materials and Methods

### Production of Lentiviral Vectors

Lentiviral vectors were produced and titered as described previously [10] and contained a CMV promoter driving constitutive expression of rat GDNF (LV-GDNF) or control vector. In contrast to GDNF, green fluorescent protein (GFP) is a foreign protein which elicits an immune response following expression in the rat sciatic nerve [23]. We therefore used a lentiviral vector encoding GArGFP, a fusion protein consisting of the Gly-Ala repeat (GAr) domain of the Epstein–Barr nuclear antigen-1, fused to the N-term part of GFP. The long alanine stretch of the GAr domain reduces the generation of antigen-linked MHC-I peptide and prevents a cytotoxic T-lymphocyte-mediated immune response against GFP, resulting in long-term expression [23], [24]. All experiments were performed using the same batches of LV-GArGFP or LV-GDNF. Titers were 9.4×10^9^ TU/ml for LV-GArGFP and 2.0×10^9^ TU/ml for LV-GDNF.

### Experimental Animals

The study was performed after its design was approved by the Animal Experimentation Committee of the Royal Netherlands Academy of Arts and Sciences. All experimental procedures were performed in accordance with the European guidelines for the care and use of laboratory animals (86\609\EEC). A total of 40 female Wistar rats (200–250 g; Harlan, Horst, The Netherlands) were used. Animals were housed under standard conditions at a 12∶12 h light/dark cycle with food and water *ad libitum*.

### In-Vitro Quantification of Transgene Expression in Relation to Viral Vector Dilution

To determine the relationship between the number of injected viral particles and the level of GDNF expression, sciatic nerve explants were injected with serial dilutions of LV-GNDF. The sciatic nerves of female rats (n = 3) were dissected at the mid-thigh level and divided into 1 cm nerve segments. A total of 15 nerve segments (n = 3 explants per concentration of vector) were injected with 1 µl of LV-GArGFP containing 9.4×10^6^ TU, or a dilution series of LV-GDNF of 2.0- 1.0- 0.5- and 0.25-×10^6^ TU per nerve segment. Injections were performed with a glass needle as described previously [23]. Individual nerve segments were cultured for 10 days post-injection in Iscove's Modified Dulbecco's Medium supplemented with 100 IU/ml penicillin, 10 ug/ml streptomycin, 2 mM glutamax, and 10% FBS. GDNF protein expression was measured by ELISA in medium samples that were collected every 24 h and were frozen and stored at −80C. After 10 days in culture all nerve pieces were snap-frozen and homogenized by grinding in ice cold mortar containing liquid nitrogen. Tissue was resuspended in 250 µl lysis-buffer (TBS containing 1% NP-40 substitute, 10% glycerol, 0.1% Tween-20, 0.5 mM Sodium Orthovanadate and roche total protease inhibitor). The GDNF ELISA was performed in high-binding ELISA plates (Nunc-Immuno Maxisorp, #439454) according to the manufacturer’s instructions (E-Max ImmunoAssay System, #G7620 Promega, Madison, USA). The absorbance was read at 450 nm using a microplate reader (Varioskan Flash, Thermo, Finland). Finally, group averages of total GDNF protein in the medium and nerve pieces were calculated per time point.

### Generation of an in vivo Gradient of GDNF in the Sciatic Nerve

All surgical procedures were performed under isoflurane anaesthesia (Isoflo, Abbott, Hoofddorp, the Netherlands). The left sciatic nerve was exposed from the sciatic notch towards the trifurcation into the tibial, peroneal and sural branch. From proximal to distal, five 1 µl injections of LV-GDNF containing 0.5- (twice), 1.0-, 1.33-, or 2.0-×10^6^ TU or titer-matched LV-GAr-GFP were made with a glass needle fixed to a 10 µl Hamilton syringe. Fast green (Sigma, Zwijndrecht, The Netherlands) was added to the viral vector solution to aid in the per-operative visualisation of the injection procedure. The most proximal injection was performed at the sciatic notch underneath the iliac bone and was marked using a 10/0 epineural suture (ethilon, Johnson & Johnson, the Netherlands). Increasing concentrations of viral vector were then injected at intervals of approximately 7–8 mm. In total, a length of approximately 4 to 4.5 cm of nerve was injected. Four weeks after surgery, animals were sacrificed by decapitation and sciatic nerves were dissected out. The epineural marking suture was used as reference, and from this marking, 5 mm nerve segments were cut, in numerical order and directly snap frozen. Nerve segments were homogenized and individual samples were analysed using a GDNF ELISA as described above. Contralateral sciatic nerves were used as non-lesioned controls.

### Ventral Root Avulsion and Implantation Surgery

A unilateral avulsion of three lumbar ventral roots (L3, L4, and L5) was performed in rats (n = 32) as described previously [23]. Briefly, under general anaesthesia using Hypnorm (Fentanyl/Fluanisone; 0.08 ml/100 g body weight, i.m. Janssen Pharmaceuticals, Beerse, Belgium) and Dormicum (Midazolam; 0.05 ml/100 g body weight s.c. Roche, Almere, the Netherlands), a unilateral laminectomy was performed and the L3, L4 and L5 ventral roots were avulsed from the spinal cord. The roots were directly implanted into the ventro-lateral part of the spinal cord at a depth of approximately 0.5 mm and fixed in place using fibrin glue (TissueCol; Baxter B.V., Utrecht, the Netherlands). Muscles and skin were closed in separate layers and animals were kept at 37°C until fully recovered. A single dose of Temgesic (Buprenorphine 0.03 ml/100 g body weight s.c., Schering-Plough B.V., Maarssen, the Netherlands) was injected for post-operative analgesia. An LV-GArGFP (n = 16) or LV-GDNF (n = 16) gradient was applied in the sciatic nerve as described above two weeks after avulsion and implantation, because a previous study showed that the first regenerating axons enter the lumbosacral plexus at this time point [3].

### Retrograde Motoneuron Tracing

To quantify the number of motoneurons that project one or more axons in the distal sciatic nerve, retrograde tracing was performed 17 weeks after avulsion and implantation (15 weeks after application of the GDNF gradient). Under deep isoflurane anaesthesia (Abbott) the sciatic nerve was transected approximately 2 cm distal from the marking suture (corresponding to segment 7) in LV-GArGFP and LV-GDNF treated rats (n = 6 for both groups). A piece of Parafilm was placed underneath the nerve to prevent tracer leakage to surrounding muscle. The proximal nerve stump was submerged in 3 µl of 5% FastBlue (FB, EMS-Chemie, Mannedorf, Switzerland) in sterile saline for 30 minutes as described previously [11]. Following tracing, the nerve was thoroughly rinsed with multiple saline washes. To allow sufficient tracer transport, animals were sacrificed 7 days post-tracing.

### Tissue Harvest and Processing

At 18 weeks post avulsion and implantation, FB traced animals were deeply anesthetized using Nembutal (sodium pentobarbital; 0.11 ml/100 g i.p. Sanofi Sante, Maassluis, the Netherlands) and transcardially perfused with cold saline, followed by 4% paraformaldehyde (PFA) in 0.1 M sodium phosphate buffered saline pH 7.4 (PBS). Lumbar spinal cords containing the L3–L6 motoneuron pool were dissected and post-fixed overnight in PFA at 4°C followed by overnight incubation in 250 mM EDTA in PBS to soften possible residual bone debris. Tissue was cryoprotected using 25% sucrose in PBS for 2 days at 4°C followed by embedding and snap-freezing in OCT Compound 4583 (Tissue-Tek; Sakura, Zoeterwoude, the Netherlands). Four series of 25 µm thick longitudinal spinal cord sections were cut on a cryostat and mounted on Superfrost Plus slides (Menzel-Gläser, Braunsweig, Germany).

Remaining animals (n = 10 LV-GArGFP, n = 10 LV-GDNF) were decapitated and sciatic nerves were exposed and freshly dissected. Using the epineural suture as reference, 5 mm nerve segments were cut and every other segment (segment 2, 4, 6 and 8) was snap frozen and processed as described above for ELISA analysis. The remaining nerve segments (segments 1, 3, 5 and 7) were post fixed in PFA overnight, cryoprotected in 25% sucrose/PBS and frozen in OCT compound. Contralateral sciatic nerves were dissected and processed identically to serve as non-lesioned controls. Four series of 30 µm transverse sciatic nerve sections were cut on a cryostat and mounted on Superfrost Plus slides. Sections were stored at −80°C until further processing.

### Staining Procedures

Immunohistochemical (IHC) staining for choline acetyl transferase (ChAT) was performed on spinal cord sections to specifically visualize motoneurons. Tissue was subjected to a 7 minute 0.1% Triton-X100 and 0.01 mg/ml proteinase K in PBS treatment for antigen retrieval followed by 10 min 4% PFA fixation. Subsequently, tissue was washed three times in PBS and blocked using blocking buffer (PBS containing 5% FCS and 0.3% Triton X-100) for 30 minutes. Sections were incubated overnight at 4°C in blocking buffer containing primary antibody against ChAT (1∶200; AB144P Chemicon, Hampshire, UK). After three washes in PBS, secondary antibody incubation was performed for 2 h (1∶800; donkey anti-goat Alexa 594; Jackson ImmunoResearch Europe Ltd., Cambridgeshire, UK) in blocking buffer. Sections were washed three times, coverslipped in Vectashield (Vector Laboratories, Burlingame, CA, USA) and processed for quantification.

Transverse sciatic nerve sections were subjected to IHC staining for ChAT as described above to quantify the total number of motor axons after 18 weeks post lesion. These sections were incubated with a biotinylated secondary antibody (1∶200; horse anti-goat Biotin; Jackson) for 2 hours, washed three times and incubated with avidin-biotin-peroxidase complex (ABC; 1∶800 PK-6100 Elite Vectastain ABC kit, Vector laboratories) for 1 h at room temperature. After 3 washes, final staining was performed using 0.035% 3′ 3′-diaminobenzamidine tetrahydrochloride (DAB) in TBS containing 0.01% H_2_O_2_ and 0.2 mg/ml (NH_4_)_2_.SO_4_.NiSO_4_ for 8 minutes. Sections were dehydrated in a graded series of ethanol, cleared in xylene and embedded in Entallan for light microscopic analysis. To obtain information about the glial response in relation to GDNF, transverse sciatic nerves were subjected to a fluorescence staining as follows: after incubation in blocking buffer for 30 min, sections were incubated overnight at 4°C in blocking buffer containing primary antibodies against S100 (1∶600; Dako, Glostrup, Denmark) to visualize Schwann cells, and GDNF (1∶500; R&D systems, Minneapolis, MN, USA) for transgene expression. After three washes in PBS, secondary antibody incubation was performed for 2 h (1∶800; donkey anti-rabbit Alexa 488 and 1∶200; goat anti-chicken biotin; Jackson) in blocking buffer. Subsequently, sections were washed three times and incubated for 30 minutes with Alexa-594 conjugated streptavidin and Hoechst (1 µg/ml; BioRad, Hercules CA). After three washes in PBS, sections were mounted in Vectashield (Vector Laboratories). Schwann cell morphology and myelination was visualised with rabbit anti-p75 (1∶250, #G323A Promega Benelux, Leiden, the Netherlands), chicken anti MBP (1∶100, AB9348 Merck Millipore, Billerica, MN, USA) and anti-neurofilament (1∶200 ms a-2H3 Developmental studies Hybridoma bank, University of Iowa, USA) with the staining procedure and secondary antibodies as described above.

### Quantification of Motoneuron Survival and Distal Outgrowth

To systematically quantify the number of surviving and retrogradely traced motoneurons present in the L3–L6 lumbar segment at 18 weeks post-lesion, every fourth section of longitudinally, horizontally cut spinal cord, spanning the entire ventral horn was stained for ChAT as described above. The total number of ChAT positive profiles in both the lesioned and contralateral side were manually counted by an observer blinded to the treatment group at a 20× magnification. ChAT positive profiles were only included if they contained a nucleus with a visible nucleolus. Simultaneously, motoneurons that were positive for the FB tracer were counted. In total, quantification was performed using approximately 15 sections per animal. Group averages of total number of traced motoneurons were calculated and plotted in a histogram. The total cell count per animal was expressed as a percentage of the contralateral side. Subsequently, group averages of both LV-GArGFP and LV-GDNF groups were calculated. Quantification was performed within 2 days after embedding in order to prevent variability due to fading of FB tracer in aqueous mounting medium during the quantification period.

### Quantification of Total Motor Axon Number and Nerve Coils

To investigate the effect of an increasing GDNF gradient on regenerating motor axons, ChAT-stained motor axons were quantified in transverse sections of the sciatic nerve as described previously [3]. Briefly, quantification was performed in nerve segments 1, 3, 5 and 7. The sections were randomized and quantification was performed by an observer blinded to the intervention group. Custom-made Image Pro Plus-based (MediaCybernetics, USA) software was used to measure the total sciatic nerve surface area using a 2.5× objective. Subsequently, a grid was placed over the nerve area and ChAT-positive axons were counted manually at 40× magnification. Using a systematic randomized sampling method, 80% of the sciatic nerve surface was quantified. The total number of fibres per nerve segment for each animal was subsequently calculated.

A semi-quantitative analysis was performed to determine the presence of dense fibre coils in nerve segments 1, 3, 5 and 7. A nerve coil was defined as a densely stained ChAT positive area containing fibres growing in a circular and or swerving orientation. Due to the local high fibre density, these areas were not included in the total fibre quantification because reliable identification of individual fibres in coils is not possible.

### Evaluation of Cellular Density in Nerve Coils

The total cell density in the sciatic nerve was measured in nerve segments 1, 3, 5 and 7 which were stained for GDNF, S100 or GFAP and Hoechst as described above. High resolution images were captured using a Zeiss axioplan microscope and a digital Evolution QEi camera (MediaCybernetics, Silverspring USA). The total nerve surface area was measured in randomized sections of the sciatic nerve that were outlined using ImagePro as described above. Nerve coils could be readily identified as areas containing a very high intensity of Hoechst signal. These areas were outlined separately. Subsequently, the total surface area covered by Hoechst-labelled nuclei was measured and expressed as a percentage of the outlined area as a measure for cell density.

### Data Analysis

All data are expressed as mean ± SEM. Statistical analysis was performed using SPSS software (v17.0; SPSS, Chicago, IL) and a value of p<0.05 was considered significant. Student’s *t*-test analysis was performed to determine statistical significance.

## Results

### Relationship between GDNF Expression Levels and Viral Vector Concentrations

**Figure 1:**
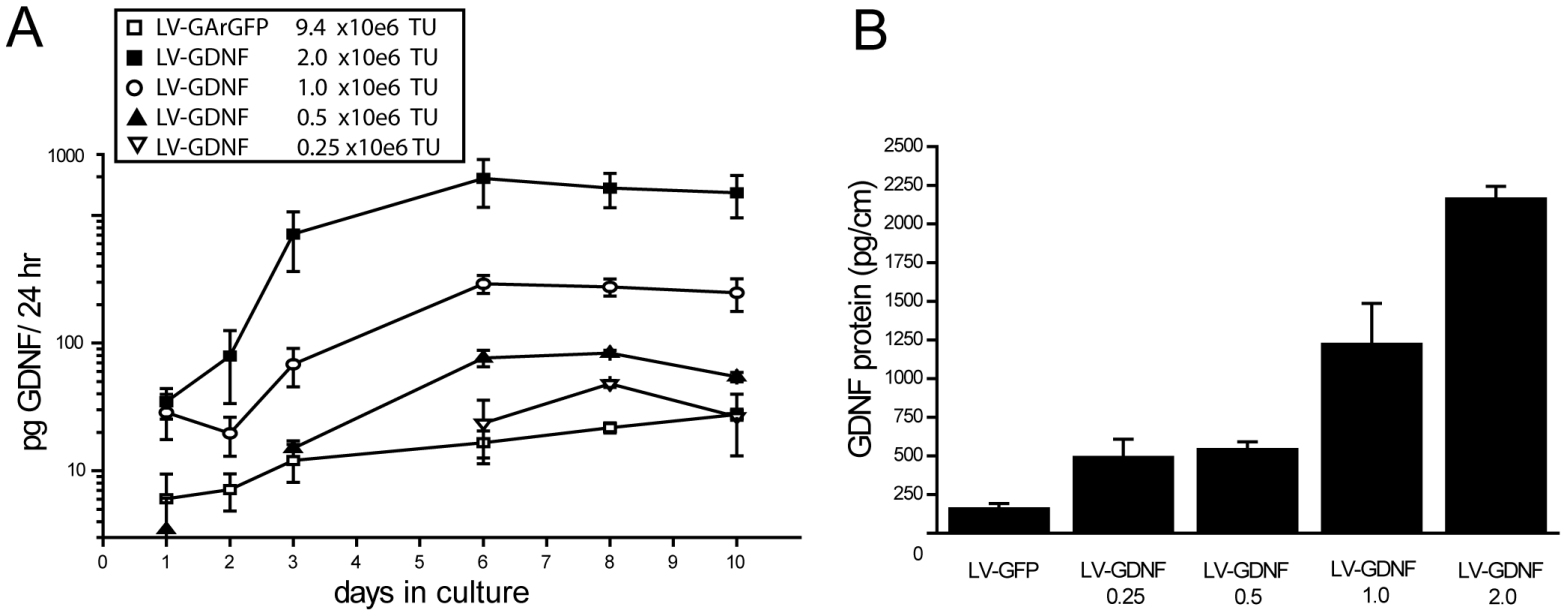
Dose dependent expression of GDNF in nerve segments transduced with increasing amounts of LV-GDNF. One centimeter segments of the sciatic nerve were injected with increasing amounts of LV-GDNF ranging from 0.25–2×10^6^ transducing units (TU) and cultured for 10 days. **A**) culture medium was harvested every 24 h and GDNF was quantified in the medium using an ELISA. GDNF levels in the medium increased from 1 to 6 days and reached a plateau between 6 and 10 days. **B**) At 10 days in culture, nerve segments were harvested and GDNF levels were quantified. GDNF levels increased with increasing doses of LV-GDNF applied to the nerve.

The purpose of this study was to determine the effect of a GDNF gradient - created via multiple lentiviral vector injections along the sciatic nerve – on axonal regeneration, coil formation and Schwann cell morphology. As a first step to achieve this goal, we determined the relationship between the amount of vector applied and the GDNF concentration in cultured nerve segments. Segments of rat sciatic nerve with a length of 1 cm were injected with increasing amounts of LV-GDNF and cultured for ten days. The injection of increasing amounts of LV-GDNF (ranging from 0.25 to2.0×10^6^ viral particles/cm nerve) resulted in an increase in GDNF secreted in the culture medium (Figure 1a). The GDNF concentration in the culture medium increased in the first few days following transduction, reaching a maximum at day 6, and remained at a plateau between day 6 and day 10. After 10 days in culture, all nerve pieces were homogenized and GDNF concentrations were measured in the nerve pieces. The total amount of GDNF protein in the nerve segments increased with increasing amounts of LV-GDNF (Figure 1B). On average, LV-GArGFP injected control nerve segments contained 160 (+/−30) pg of endogenous GDNF. GDNF expression had increased more than tenfold to an average of 2164 (+/−78) pg per nerve in the group receiving the highest concentration of LV-GDNF.

### Creation and Characterisation of a GDNF Gradient in vivo

**Figure 2:**
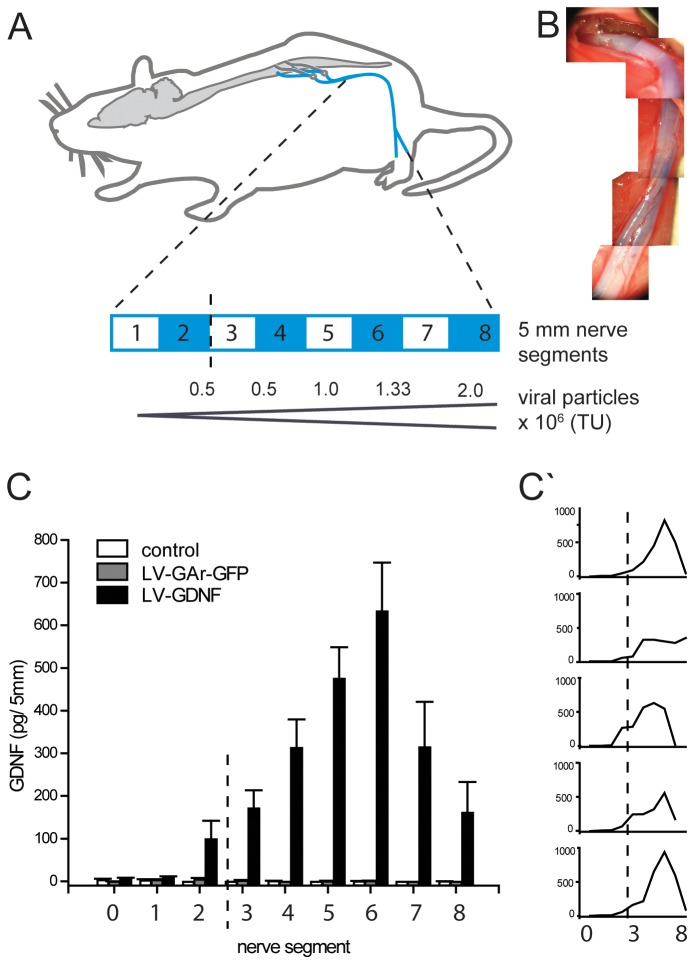
Creating a GDNF gradient in the intact rat sciatic nerve by injecting increasing amounts of LV-GDNF along the nerve. **A**) Overview of the spinal cord, lumbar ventral roots and the sciatic nerve with a schematic enlargement of the nerve that was injected with increasing amounts of LV-GDNF ((0.25–2×106 TU) or LV-GArGFP at 5 locations at 7 to 8 mm intervals. After four weeks, the sciatic nerve is removed and divided in 5 mm segments for GDNF quantification. The blue-white bar depicts the position of each harvested segment, the dotted line marks the epineural suture visible in (B). The approximate vector injection site is shown below the blue-white bar. **B**) Per-operative photographs of the sciatic nerve after injection of LV-GDNF mixed with Fast green (a dye) to visualize the injection. An epineural suture, marking the most proximal injection, is visible in the proximal end of the nerve. Note the even spread of the viral vector solution along the entire length of the nerve. **C**) Total GDNF protein in homogenized 5 mm nerve segments, 4 weeks after injection. A clear gradient of increasing GDNF levels is present in the LV-GDNF group, but not in the LV-GArGFP or non-injected control groups. The dotted line corresponds to the site of the epineural suture visible in (B). **C’**) GDNF protein levels in individual animals: although there is a slight variation in the location and level of GDNF expression, a gradient is present in the nerve of each animal.

To investigate the conditions for creating a GDNF gradient in vivo we first injected increasing amounts of LV-GDNF in the intact sciatic nerve from the sciatic notch towards the trifurcation into the tibial, peroneal and sural branch (Figure 2AB). Five injections at 7 to 8 mm intervals resulted in the transduction of approximately 4 cm of nerve. Four weeks after the injection of the lentiviral vector, the nerve was dissected and GDNF concentrations were determined in each of the eight nerve segments. This revealed a reproducible proximo-distal gradient of increasing GDNF expression in all animals (Figure 2C). There was a clear linear correlation between vector concentration and GDNF levels in the nerve: the highest levels of GDNF were found in nerve segment 6, which corresponded to the segment injected with the second highest titer, 1.3×10^6 ^TU. This segment contained on average 633 (+/−113) pg GDNF, a substantial increase compared to both LV-GArGFP injected and control nerves. In each individual animal, a clear gradient over several centimeters was detectable (Figure 2C’). GDNF levels in both LV-GArGFP injected and control nerves were low (<10 pg) and did not display a gradient.

### LV-mediated GDNF Gradients Remain Present up to 17 Weeks after Injury

**Figure 3:**
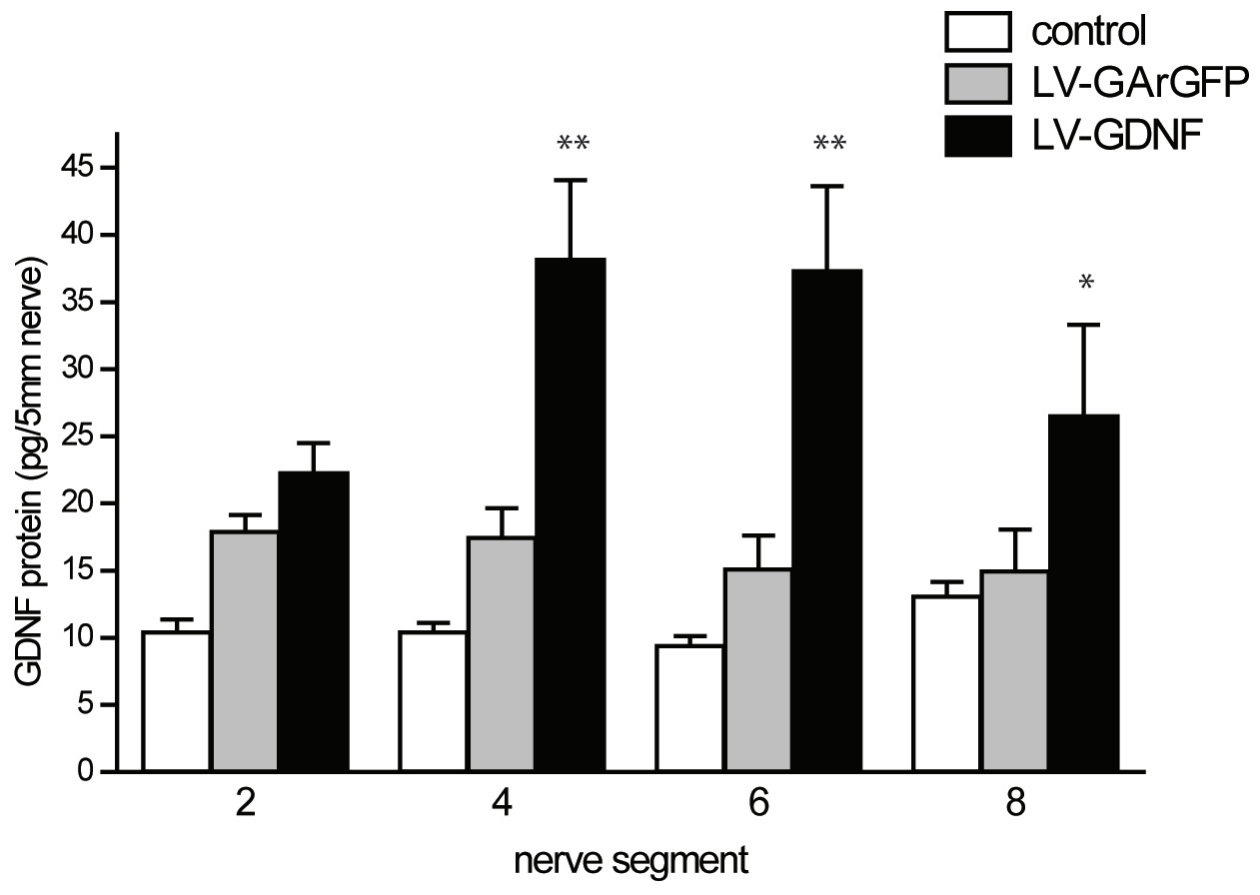
Long term expression of GDNF *in vivo* in a lesioned sciatic nerve. LV-GDNF or LV-GArGFP control vector was injected as described in Figure 2, two weeks after avulsion and reimplantation of the ventral roots L3, L4 and L5. The amount of GDNF was measured in nerve segments 2, 4, 6 and 8 (see blue-white bar in Figure 2) at 17 weeks after lesion. GDNF protein levels in the distal nerve segments are significantly higher in LV-GDNF injected animals in segments 4, 6 and 8; **p<0.001 vs LV-GArGFP, *p<0.05 vs control (non-lesioned nerve).

We next investigated whether it is possible to create a GDNF gradient in the sciatic nerve after ventral root avulsion and reimplantation. Following ventral root avulsion, implantation and lentiviral vector injection, a clear GDNF gradient was present at 15 weeks in the LV-GDNF injected nerves but not in LV-GArGFP injected nerves. The GDNF concentration was significantly increased in the middle and distal segments of the LV-GDNF injected animals (P<0.001 and P<0.05, respectively; Figure 3). Compared to the data from the *in vitro* study and the injected intact nerve at four weeks, GDNF levels were much lower, reaching a maximum of 38 (+/−6) pg in the 5 mm middle segment. As in the intact nerve experiment, GDNF levels in both LV-GArGFP and control nerves were equally low and did not increase along the length of the sciatic nerve.

### Dose Dependent Effect of GDNF on Nerve Coil Formation

**Figure 4:**
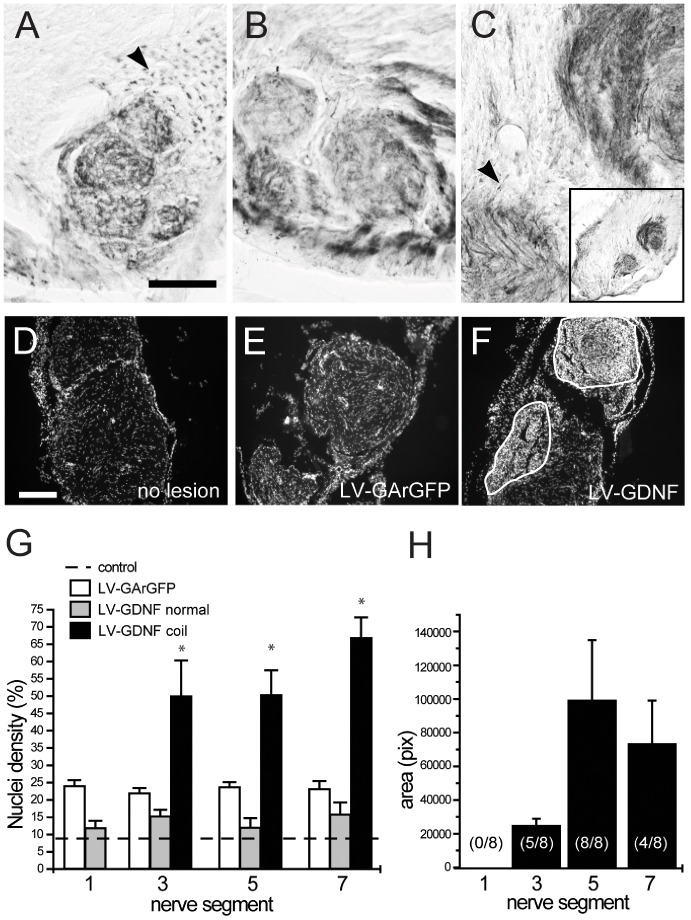
Injection of LV-GDNF leads to both increased axon numbers and formation of nerve coils with increased cellular density. **ABC**) Representative ChAT staining of motor fibers in three individual LV-GDNF treated animals at segment 5. These coil structures occurred only in LV-GDNF treated animals, and the number of coils increased towards the segments containing high levels of GDNF. Inset: lower magnification of the same nerve to show the size of the nerve coils relative to the cross-section of the nerve. **DEF**) Cellular density visualised by Hoechst nuclear staining in transverse sections of a non lesioned (D), LV-Gar GFP injected (E) and LV-GDNF (F) treated nerve. LV-GDNF injected nerves contain areas with an increased cellular density [outline in (F)]. Scale bar in A–F: 250 µm **G**) Cellular density expressed as the percentage of the surface covered by cell nuclei. The cellular density inside the nerve coils after application of LV-GDNF is significantly higher in segments 3, 5 and 7 than in the LV-GArGFP group, *p<0.02, but the cellular density in coil-free areas is similar. **H**) Quantification of the size of the nerve coils in LV-GDNF treated animals. The size of the coil corresponds to the amount of injected vector (compare Figure 2) and GDNF (compare Figure 3). The number in brackets indicates how many animals in each group had a nerve coil in this segment (no coils were present in segment 1).

Consistent with previous studies, LV-mediated overexpression of GDNF led to the formation of nerve coils. (Figure 4A–C). These nerve coils were composed of large numbers of motor axons and densely packed small cells, as revealed by ChAT and Hoechst nuclear staining (Figure 4D–G). No nerve coils were observed in any of the LV-GArGFP injected animals. In the nerves of the LV-GArGFP group (Figure 4FG), the nuclear density was uniformly higher (p<0.0001) than in uninjured control animals, presumably as a result of injury-induced Schwann cell proliferation (Mirsky et al., 2008).

The gradually increasing LV-mediated GDNF expression along the length of the nerve enabled us to study the effect of increasing GDNF protein levels on coil formation in the nerve. First, coil formation already occurred in nerve segments with relatively low levels of GDNF expression and was not prevented by creating a GDNF-gradient in the nerve. Second, in LV-GDNF treated animals, the increased cellular density was restricted to the nerve coils. There was no increase in cellular density in the nerves of LV-GDNF treated animals (compared to LV-GArGFP) when the nerve coils were excluded from the analysis. In other words: in coil-free areas the cellular density was similar to that in control nerves (Figure 4FG). Finally, there was a clear correlation between the levels of GDNF expression and the formation of the coils: no coils were seen in segment one, but coils were universally present in segment five (Figure 4H). The size of the nerve coils clearly correlated with the levels of GDNF expression: the nerve coils were largest in the nerve segments with the highest levels of GDNF, where they occupied a substantial proportion of the cross-sectional area of the nerve (Figure 4FH).

### Cellular Morphology is Disrupted and Myelination is Impaired in Nerve Coils

**Figure 5:**
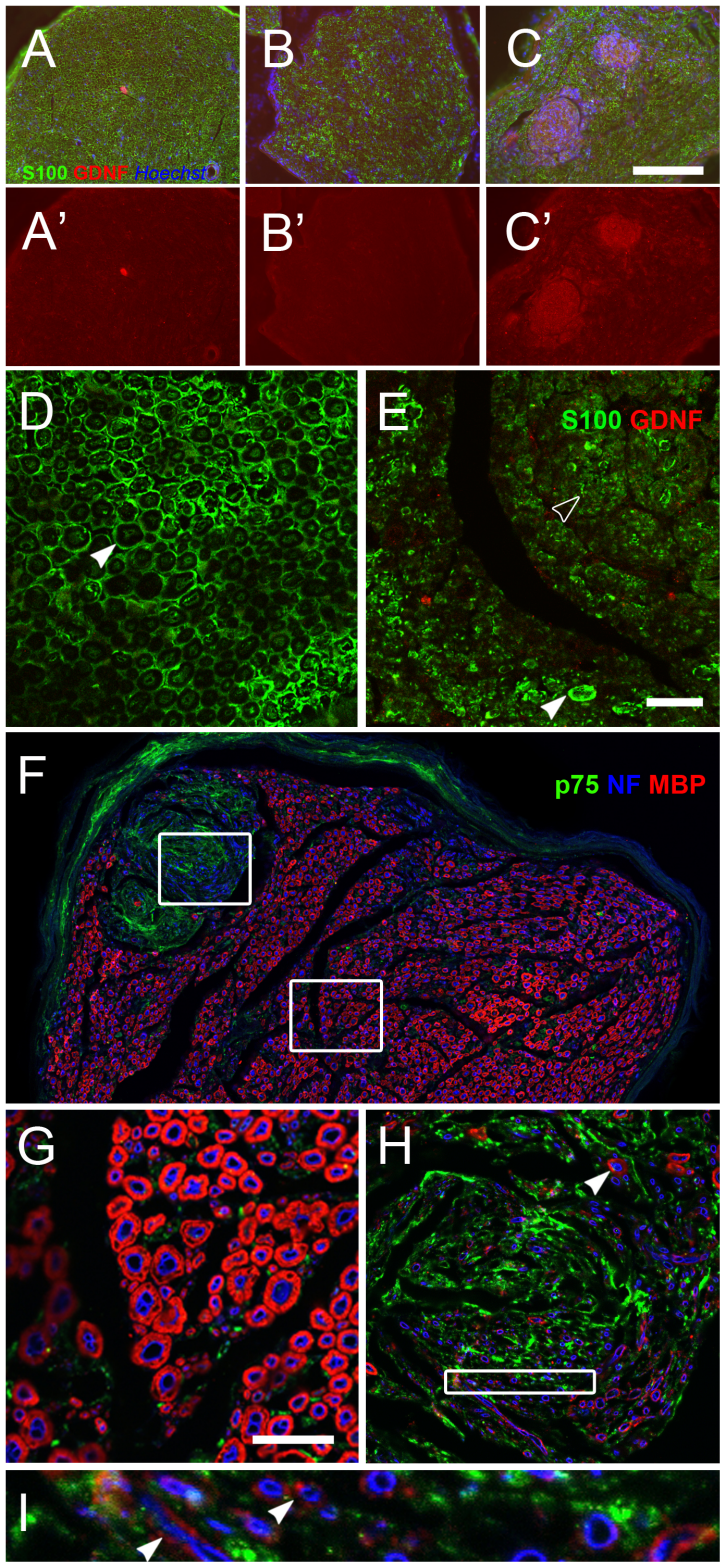
Schwann cell morphology and myelination are affected in the nerve coils. **ABC**) Transverse section of non-lesioned (A), LV-GArGFP injected (B) and LV-GDNF injected (C) nerves, stained for Schwann cells (green), GDNF (red) and cell nuclei (blue). Non-lesioned and control-injected nerves show a homogenous distribution of Schwann cells. In LV-GDNF treated nerves, there are nerve coil areas with an increased density of Schwann cells. **A’B’C’**) Same images as ABC, but with green and blue channels removed to visualise GDNF staining. In intact and lesioned control nerves, GDNF immunoreactivity does not exceed background levels. In LV-GDNF treated animals, high GDNF immunoreactivity is limited to the nerve coils. **DE**) In intact control nerves (D), and outside the coils in LV-GDNF treated nerves (E) Schwann cells have the normal, ring-like morphology (white arrowheads). This morphology appears to be disrupted in the coils. **F**) Strong p75 immunoreactivity (green) was observed inside nerve coils, indicating the presence of large numbers of immature Schwann cells. The majority of axons (blue) inside the nerve coil are not myelinated (myelin basic protein, red). **GHI**) Higher magnifications of (F). A small number of axons inside the nerve coil appear to be myelinated, but the myelin sheath was thin and only weakly immunoreactive for myelin basic protein. Scalebar in C; 200 um, Scalebar in E and G; 25 um.

The cellular composition of the nerve coils and their relationship to LV-mediated overexpression of GDNF in the nerve was studied in more detail by immunohistochemistry for S100, p75, MBP and GDNF (Figure 5). In the intact and the lesioned, control vector-injected sciatic nerve, myelinating S100-positive Schwann cells could easily be distinguished (Figure 5ABC) and GDNF immunoreactivity did not exceed the background signal (Figure 5A’B’). In contrast, high levels of GDNF immunoreactivity were observed in individual nerve coils (Figure 5C’). These coils were immunoreactive for the Schwann cell marker S100, but the S100 staining of the Schwann cells in the coils was quite diffuse (Figure 5C). The morphology of the Schwann cells in the coils was clearly disrupted (Figure 5B and D, arrows). The Schwann cells in the coils were densely packed together and it was very difficult to identify individual cells. Outside the coils, S100 staining in nerves of LV-GDNF treated animals (Figure 5D and E, white arrowheads) were visible in typical ring-like structures around axons, consistent with the morphology of myelinating Schwann cells (Figure 5E, black arrowheads). Strong p75 immunoreactivity was observed inside nerve coils, indicating the presence of a large number of immature, non-myelinating Schwann cells (Figure 5F) [25]. Outside the nerve coils, p75 immunoreactivity was low (Figure 5G), similar to intact nerves and the nerves of lesioned, LV-Gar GFP injected control animals (data not shown). Whereas the large majority of axons outside the nerve coils had thick myelin sheaths (Figure 5G), most nerve fibres inside the coil areas were not myelinated (Figure 5H). A small number of axons inside the nerve coil did appear to be myelinated (Figure 5I, arrowheads), but the myelin sheath was thin and only weakly immunoreactive for MBP. Taken together, these results indicate that the interaction between regenerating axons and Schwann cells is disrupted and myelination is impaired by the continuous expression of high levels of exogenous GDNF in the nerve coils.

### The Effect of a GDNF-gradient on Motor Axon Outgrowth in Coil-free Areas

**Figure 6:**
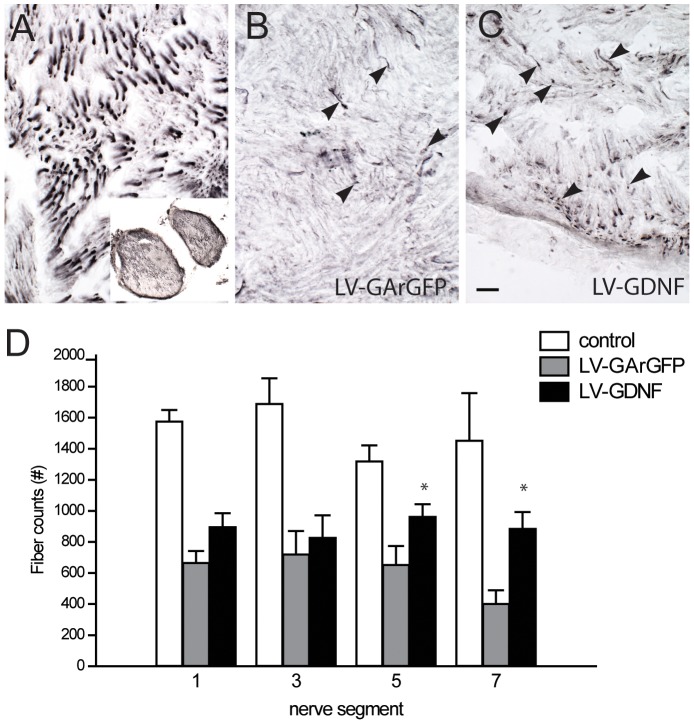
The LV-GDNF gradient leads to significantly more motor axons in the distal sciatic nerve 17 weeks after ventral root avulsion and reimplantation. **ABC**) ChAT staining of transverse sections of a non-avulsed sciatic nerve (a) shows thick motor axons within their nerve fascicles. Inset: overview of transverse area of the entire nerve. In avulsed and LV-GArGFP (B) or LV-GDNF (B) injected nerves, there are fewer ChAT positive motor axons (arrowheads), they are thinner and lack the longitudinal alignment of unlesioned axons. Scale bar in C: 25 um. **D**) The number of axons in lesioned animals is lower than intact control nerves. Whereas the number of motor axons declines in the distal segments of the nerve in LV-GArGFP treated animals, the number of axons remains constant after LV-GDNF treatment. In the 5^th^ and 7^th^ segment, the number of fibers is significantly higher after application of the GDNF gradient, *p<0.05 vs LV-GArGFP. Note that coil areas were excluded from fibre quantification.

At 17 weeks after ventral root avulsion and implantation numerous regenerating motor axons were observed in the coil-free areas of the denervated sciatic nerve. Compared to motor axons in the control, unlesioned nerve (Figure 6A), these regenerating axons had a smaller calibre and were unevenly distributed through the nerve (Figure 6BC). To determine whether GDNF promoted the growth of axons in the coil-free areas of the nerve, ChAT-stained motor axons were counted in transverse sections in 4 nerve segments. With this approach, axon counts were obtained at regular distances along the entire length of the GDNF-gradient. Nerve coil areas were excluded from fibre quantification in LV-GDNF treated animals. In intact control nerves, motor axons could readily be identified by their intense ChAT staining (Figure 6A). On average, a segment of the intact nerve contained 1510 (+/−89) motor axons. In LV-GArGFP injected animals, there was a clear decline in the number of motor axons towards the most distal portion of the nerve with an average of 654 fibers in segment 5 and 403 fibres in segment 7 (Figure 6D). In LV-GDNF treated nerves there were on average 963 and 886 fibres in nerve segments 5 and 7, respectively. Thus, in LV-GDNF treated animals, the number of ChAT positive fibres did not decline in more distal nerve segments and was significantly higher than in LV-GArGFP animals (p<0.05).

**Figure 7:**
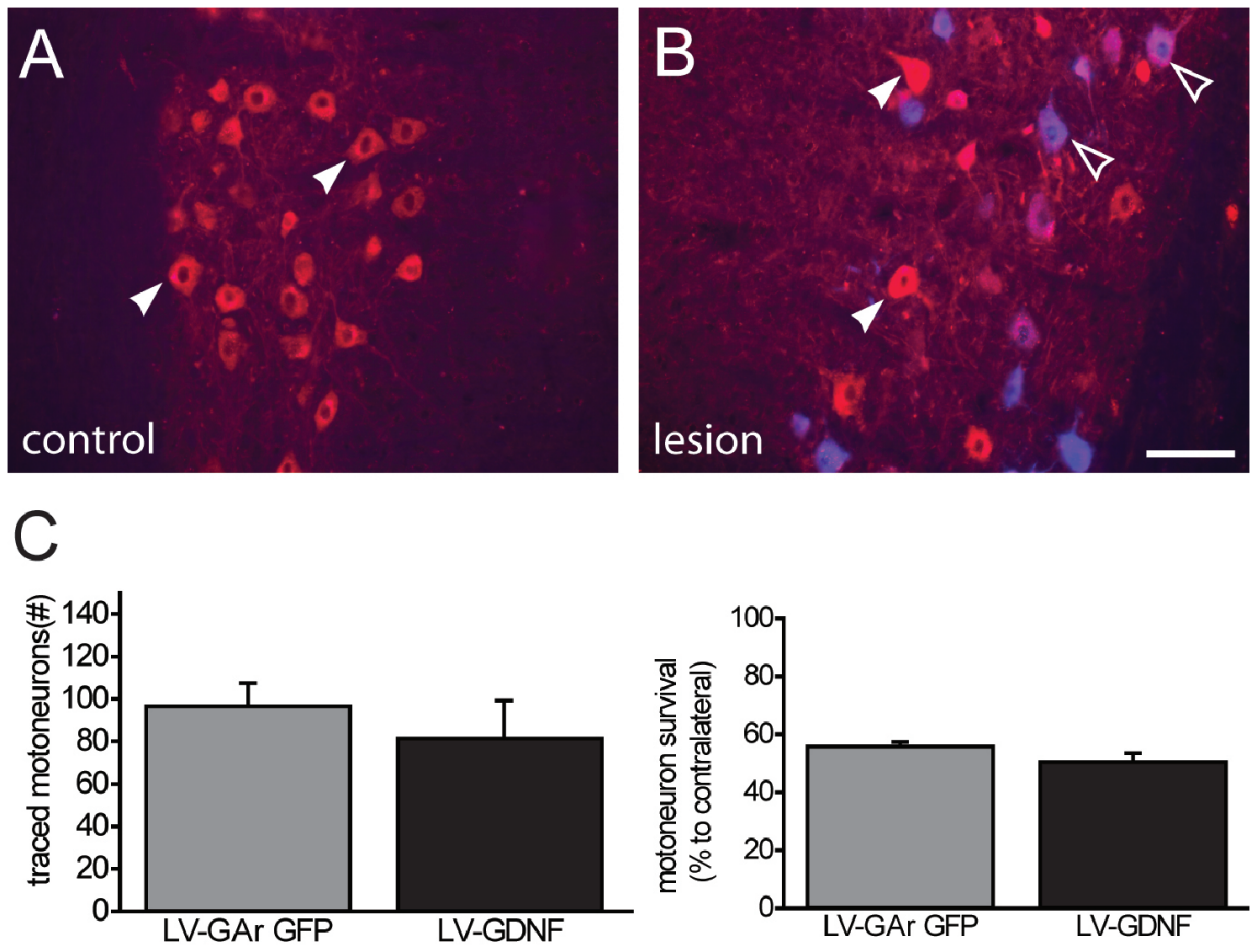
The LV-GDNF gradient does not increase the total number of motoneurons that reach the distal nerve. Regenerated motoneurons were retrogradely traced at the the mid-thigh level (corresponding to segment 6 in Figure 1), 16 weeks after lesion. A) Non-lesioned motoneuron pool stained for ChAT (red), demonstrating normal motoneuron morphology (arrowheads). B) Lesioned motoneuron pool containing retrogradely labeled neurons (blue, open arrowheads) and non-traced motoneurons (closed arrowheads). C) Left panel: the number of in the number of retrogradely labeled (i.e., regenerated) motoneurons does not differ between LV-GArGFP and LV-GDNF groups. Right panel: the number of visible motoneurons is approximately 50% of the contralateral side in both groups (right panel). Scale bar in B: 100 µm.

The increased fibre counts in the distal segments of LV-GDNF treated animals can be explained by either 1) enhanced distal axon outgrowth of individual motor axons or 2) increased sprouting of motor axons at the site of increased GDNF expression or 3) a combination of 1 and 2. To distinguish between these possibilities, the number of motoneurons that successfully extended an axon down the nerve was quantified through the application of a retrograde tracer to the sciatic nerve at the site with the highest average GDNF expression (Figure 7AB). The number of retrogradely traced motoneurons did not differ significantly between the LV-GDNF and LV-GArGFP treated animals (Figure 7C). This indicates that the higher axon counts in LV-GDNF treated animals were the result of local sprouting and do not reflect enhanced long-distance regeneration of motor axons. Motoneuron survival was quantified by counting ChAT positive profiles as a percentage of the contralateral motoneuron pool. The average number of surviving motoneurons 17 weeks after ventral root avulsion and reimplantation was 53% (Figure 6C) and did not differ between LV-GArGFP and LV-GDNF treated animals, indicating that LV-GDNF does not enhance motoneuron survival when applied to the distal part of the sciatic nerve. In both groups, 14% of surviving motoneurons contained the retrograde tracer, indicating that these motoneurons had regenerated towards the site of tracer application.

## Discussion

In this paper, we demonstrate that lentiviral vectors can be used to create a 4 cm long GDNF gradient in the intact and lesioned rat sciatic nerve. We applied this technique to test the hypothesis that such a gradient prevents GDNF-mediated nerve coil formation and stimulates the extension of axons to more distal nerve segments. However, we show that nerve coil formation occurs even in the presence of a gradient and that both the number and the size of the nerve coils are directly related to local GDNF levels. In these coils, Schwann cell density is increased, their morphology is disrupted and myelination of axons is severely impaired. The total number of regenerated and surviving motoneurons is not enhanced after the distal application of a GDNF gradient, but increased sprouting does result in higher motor axon numbers in coil-free areas in the distal segment of the sciatic nerve.

### Creation of a Long Distance Neurotrophic Gradient in the Peripheral Nerve

The application of exogenous neurotrophic factor gradients has been reported previously in the injured spinal cord [18], [19] and in artificial nerve conduits [17]. This is the first study that describes the effects of a neurotrophic factor gradient in a lesioned peripheral nerve. In the brain GDNF can diffuse up to 11 mm after injection of the protein [26], [27]. Using these findings as a guideline, we injected a lentiviral vector encoding GDNF at 7 to 8 mm intervals and hypothesized that this would be sufficient to create an evenly distributed GDNF-gradient, especially since a single injection transduces cells over a distance of several mm [11], [23]. Indeed, the ELISA data show that GDNF protein levels increased gradually over a 4 cm segment of the nerve. Unexpectedly, the most distal segments showed lower levels of GDNF despite having received injection of LV-GDNF at the highest titer. The most distal injection site corresponded to a segment of the sciatic nerve directly proximal to the knee, where it splits in the peroneal and tibial nerves. This anatomical feature may have hampered the spread of the viral vector distally in some animals. Figure 2C’ indeed shows that GDNF levels were relatively variable in the most distal segment (segments 7 and 8), but more uniformly elevated in segment 6, resulting in a higher average GDNF level.

### A Neurotrophic Gradient does not Prevent Nerve Coil Formation

At 15 weeks after the injection of the lentiviral vector, GDNF expression is specifically associated with the nerve coils that have formed in the nerve. Nerve coil formation was never observed in LV-GArGFP injected nerves, so these coils are not formed as a result of the injection of the lentiviral vector itself. The highly focalized expression of GDNF within some regions of the nerve is likely caused by the combined effect of the injection technique and the anatomical properties of the peripheral nerve on the spread of the viral vector during injection. The vector is applied to the nerve by inserting a glass capillary in a nerve fascicle and releasing the vector solution as the needle is slowly retracted. This is likely to result in the selective transduction of longitudinal bands of Schwann cells near the needle tract.

Schwann cells express the receptors for GDNF [21], [28] and GDNF has been shown to promote Schwann cell proliferation in vitro in the presence of axons [28], [29]. In uninjured nerves, high exogenous doses of GDNF also cause a twenty-fold increase in Schwann cell proliferation in vivo [22]. Similarly, and in agreement with previous studies [10], [17], we show that Schwann cell density is significantly increased in the nerve coils, presumably as the result of increased Schwann cell proliferation. Another possibility is that Schwann cells migrated towards areas of high GDNF expression. However, a recent study suggests that GDNF signaling does not play a major role in Schwann cell migration [30], suggesting that increased migration of Schwann cells towards sites of GDNF expression was not a major contributing factor to the formation of coils.

### LV-GDNF Transduction Negatively Affects Schwann Cell Maturation and Myelination

Schwann cells inside nerve coils do not have the elongated, ring-like morphology of myelinating Schwann cells and they express high levels of p75, whereas the motor axons in the nerve coils are not or very poorly myelinated. Together, these findings show that transduction of Schwann cells with LV-GDNF negatively affects their maturation and their ability to myelinate regenerating axons [25]. This conclusion is in agreement with the results of a recent study which showed that myelination is impaired in nerve grafts seeded with GDNF-overexpressing Schwann cells compared to naïve Schwann cells [12].

In contrast, high levels of systemically delivered exogenous GDNF have previously been shown to enhance the myelination of axons in vivo and result in the myelination of fibres that are normally not myelinated [22]. In the study by Hoke et al, GDNF was administered systemically to mice without applying a nerve injury, i.e. to Schwann cells in a quiescent, myelinating state. Following nerve root avulsion Schwann cells dedifferentiate and switch to a non-myelinating, outgrowth-promoting phenotype [31]. The current observations suggest that the overexpression of GDNF in a lesioned nerve specifically inhibits the phenotypic switch from a growth-promoting, dedifferentiated to a myelinating, mature state. Furthermore, there was a difference in the mode of GDNF application. While it was delivered systemically in the previous study, it was applied locally by means of lentiviral transduction in our study, resulting in high intracellular levels in transduced cells.

### Neurotrophic Effects of GDNF: Sprouting, not Regeneration or Survival

Significantly increased sprouting of motoneurons was observed in coil-free areas along the GDNF mediated gradient. The effects of GDNF on axonal branching have been described previously in the central nervous system and at the neuromuscular junction (NMJ). Centrally, injury-induced sprouting of dopaminergic cells is mediated in part by GDNF [32] and LV-mediated overexpression leads to aberrant sprouting of dopaminergic cells [33]. Peripherally, overexpression of GDNF in skeletal muscle causes hyperinnervation at the NMJ [34], increases the number of NMJs and impairs the process of synapse elimination, resulting in hyper-innervated end plates [35]. Furthermore, treatment with exogenous GDNF causes continuous synaptic remodeling and axonal branching at the NMJ [36]. Our data show that GDNF is also a potent inducer of axon sprouting in the injured peripheral nerve.

In contrast to previous studies [10], there was no effect of GDNF on motoneuron survival in this study. In the previous study, LV-GDNF was applied directly after injury in the reimplanted spinal roots in very close proximity to the site of injury. In the present study, there was a distance of several cm between the area of transgene expression and the site of injury. It is technically challenging to inject the proximal part of the sciatic nerve because it runs underneath the pelvic bone after exiting the spinal canal. This distance may have limited any direct GDNF-mediated effects on the survival of avulsed motoneurons. Indeed, retrograde tracing showed that the percentage of surviving motoneurons reaching the level of maximum transgene expression at 17 weeks was only 14% in both in the LV-GDNF and the control group. Consequently, the majority of injured motoneurons did not reach the area of maximum transgene expression, limiting any potential effects on survival.

Furthermore, the viral vector was injected two weeks after the initial injury. This decision was made because a previous study showed that at this time point the first regenerating nerve fibres start to enter the lumbosacral plexus while endogenous GDNF levels in the distal sciatic nerve start to decline [3]. However, this delay may have caused some motoneurons to atrophy before LV-GDNF was applied [3].

Whereas in previous studies clear evidence of trapping of regenerating axons after application of LV-GDNF was observed based on retrograde tracing, the number of retrogradely labeled motoneurons was not decreased in the present study. In the previous study, however, the retrograde tracer was applied [11] *distal* from the site of viral vector-mediated GDNF expression. Here, we applied the retrograde tracer at the site of maximum GDNF expression (and at a location where abundant nerve coil formation was observed) because we aimed to study the effect of GDNF on the regeneration of motor neurons.

### Regulation of GDNF Expression may Prevent Unwanted Side-effects

In summary, Lentiviral vector-mediated overexpression of GDNF exerts multiple effects on both motoneurons and Schwann cells in the lesioned peripheral nerve. The axons of injured motoneurons form nerve coils when they enter areas containing high levels of GDNF. We and others have previously shown that this results in impaired long distance regeneration [10]–[12]. By applying a gradient of increasing amounts of vector, we show in this study that this nerve coil formation is correlated to the GDNF concentration in the nerve, which in turn depends on the amount of vector injected. However, small nerve coils are even formed in nerve segments with low amounts of transgenic GDNF expression, suggesting that the therapeutic window for overexpression of GDNF is relatively small.

Future studies will have to focus on exploiting the beneficial properties of GDNF, in particular its ability to prevent injury-induced motoneuron atrophy, while avoiding its negative effects on both motor axons (trapping) and Schwann cells (maturation an myelination). Short-term, controlled expression of GDNF, with the use of a non-immunogenic vector with regulatable transgene expression [37], could temporarily boost the regenerative process without impairing long distance outgrowth, Schwann cell maturation or axon myelination.
